# Development and Evaluation of a Cognitive Training Game for Older People: A Design-based Approach

**DOI:** 10.3389/fpsyg.2017.01837

**Published:** 2017-10-17

**Authors:** Ming-Hsin Lu, Weijane Lin, Hsiu-Ping Yueh

**Affiliations:** ^1^Department of Bio-Industry Communication and Development, National Taiwan University, Taipei, Taiwan; ^2^Department of Library and Information Science, National Taiwan University, Taipei, Taiwan; ^3^Department of Psychology, National Taiwan University, Taipei, Taiwan

**Keywords:** design-based research, elderly adult, cognitive training, mobile game, app

## Abstract

In the research field of cognitive aging, games have gained attention as training interventions to remediate age-related deficits. Cognitive training games on computer, video and mobile platforms have shown ample and positive support. However, the generalized effects are not agreed upon unanimously, and the game tasks are usually simple and decontextualized due to the limitations of measurements. This study adopted a qualitative approach of design-based research (DBR) to systematically review and pragmatically examine the regime, presentation and feedback design of a cognitive training game for older adults. An overview of the literature of cognitive aging and training games was conducted to form the theoretical conjectures of the design, and an iterative cycle and process were employed to develop a mobile game for older adults who are homebound or receiving care in a nursing home. Stakeholders, i.e., elderly users and institutional administrators, were invited to participate in the design process. Using two cycles of design and evaluation, a working prototype of an iPad-based app that accounted for the needs of elderly adults in terms of form, appearance and working function was developed and tested in the actual contexts of the participants' homes and an assisted living facility. The results showed that the cognitive training game developed in this study was accepted by the participants, and a high degree of satisfaction was noted. Moreover, the elements of the interface, including its size, layout and control flow, were tested and found to be suitable for use. This study contributes to the literature by providing design suggestions for such games, including the designs of the cognitive training structure, interface, interaction, instructions and feedback, based on empirical evidence collected in natural settings. This study further suggests that the effectiveness of cognitive training in mobile games be evaluated through field and physical testing on a larger scale in the future.

## Introduction

The number of elderly people in the world is increasing rapidly. Taiwan will have become an aged society by 2018 and a super-aged society by 2025 (National Development Council, 2016). To cope with aging populations, governments and institutions around the world have proposed various guidelines, such as successful aging (Rowe and Kahn, 1997), active aging (Foster and Walker, 2015) and healthy aging (World Health Organization, 2015). The purpose of these guidelines is generally to propose how elderly people's cognition, capacity and physical function can be maintained, to encourage their social participation and to emphasize the competence and knowledge that older people possess. The idea of cognitive aging has gained attention as domain-specific, age-related cognitive changes that—at a minimum—affect attention, executive functioning, memory, language and visuospatial function, as indicated by neurochemical theory, localized theory and process theory (Dempster, 1992; Salthouse, 1996; West, 1996; Volkow et al., 1998; Bäckman et al., 2006; Clay et al., 2009; Drag and Bieliauskas, 2010; Craik and Rose, 2012; Turgeon et al., 2016). Normal aging leads to changes in inhibition and selective attention. These changes make older people more susceptible to the negative effects of divided attention, and task-switching is thus harder to perform (Kray and Lindenberger, 2000; Mayr and Kliegl, 2000). One significant finding of related studies is that the aging-related decreases in working memory affect executive functioning. Executive functioning is a high-order cognitive structure that uses and organizes large amounts of information to support goal-directed behavior (Dobbs and Rule, 1989; Wang et al., 2011). The lower speed of time perceived by the elderly also affects their executive performance (Turgeon et al., 2016). The age-related deficiency of executive function is a strong predictor of functional impairment in the elderly living in communities or assisted-living facilities (Wang et al., 2011). In addition, the age-related memory decline has a particular effect on recollection ability during information finding and retrieval (Park et al., 2002). Recall is more difficult than recognition for elderly people, and their semantic memory appears to have a deficit (Naveh-Benjamin, 2000). However, the related cues of familiarity, auditory context, and external context can help the recollection process of older people (Park et al., 1990; James and Burke, 2000; Bastin and Van der Linden, 2003). Another change is the age-related decline in language ability performance, which is affected by inhibition capability, working memory and the recollection process. This decline makes it more difficult for elderly people to understand sentences and recall text because of the great syntactic complexity these tasks involve (Gold and Arbuckle, 1995; DeDe et al., 2004). According to previous studies, visuospatial abilities such as visuospatial attention, memory and orientation are also affected by age. Integrative visuospatial tasks such as problem solving (e.g., route-finding) and mental transformation (e.g., map reading) are sensitive to age and associated with executive function. In addition, testing visuospatial access can more efficiently detect Alzheimer's patients at the early stage of cognitive decline than can testing verbal access (De Federicis et al., 2016).

**Figure 1:**
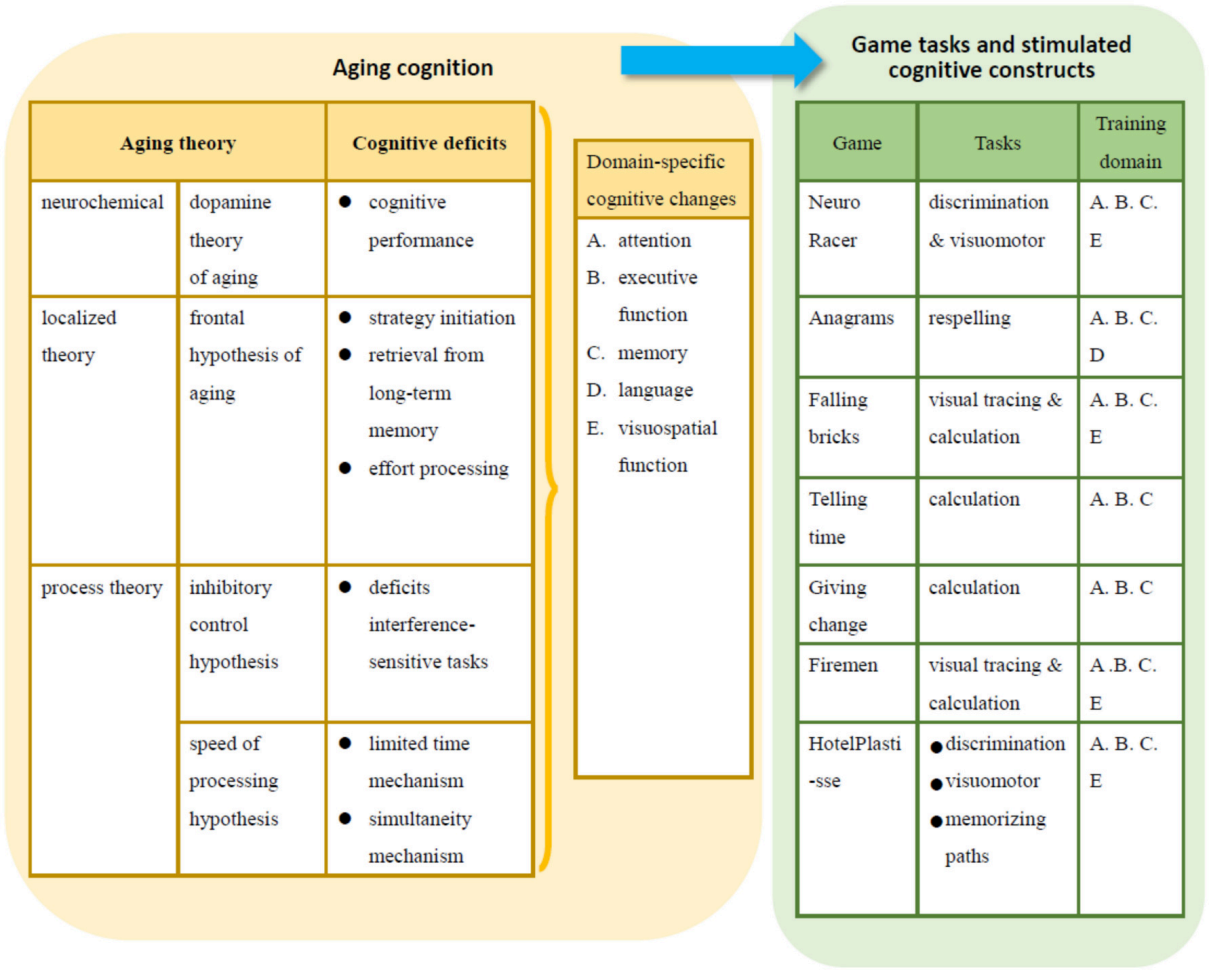
Aging cognition and cognitive training structure of related games.

Pursuing these questions, an increasing number of studies have demonstrated that games can have a positive impact on senior citizens and can provide them with cognitive training (Flynn et al., 2007; Ackerman et al., 2010; Calvillo-G'amez et al., 2010; Anguera et al., 2015). These empirical studies have proposed certain components that make cognitive training games successful, such as training content comprised of customized and adaptive tasks (Lachman, 2006), well-designed interaction via friendly interfaces (Schmiedek et al., 2010), and the accessibility of the devices (Lindenberger et al., 2008; Schmiedek et al., 2010). While these components are important references for the formation of design guidelines, their effects on cognitive training are not agreed upon unanimously (Owen et al., 2010). The rapid development of the devices and users' adjustability to the affordance of the devices introduce great variety and difficulty to systematic and empirical investigations (Van Muijden et al., 2012; Anguera et al., 2015). In addition to the employment of advanced methods and techniques to collect neural and behavioral evidence, a few, yet critical, attempts have also been made at the situated effect of the training context (Rush et al., 2006; Trefry, 2010; Binder et al., 2015). The results have suggested a more comprehensive range of research for cognitive training game studies. Figure 1 summarizes the relationships among aging theory, age-related cognitive changes and associated cognitive training games, and their effects revealed by the related studies.

An overview of the literature has shown that designing technology products with a friendly interface for elderly people requires that designers consider the declines in older users' perception, sensation, motion and cognition (Drag and Bieliauskas, 2010; Lu and Yueh, 2015). For instance, considering older adults' declines in perception and sensation, decisions to use visual presentations such as reflective, decorative or animated images should be made cautiously (Namba et al., 1995; Green and Bavelier, 2003). Other design principles regarding auditory feedback (Charness and Jastrzembki, 2009; Fisk et al., 2009), haptic feedback (Lee and Kuo, 2001; Harada et al., 2013), and memory assistance (Ferreira and Pithan, 2005; Fisk et al., 2009; Sauve et al., 2015) have also been reported in related studies. According to this review, the present study proposes five principles that should be considered during the development of the interface and interaction in a digital game for the cognitive training of elderly people: (1) Provide clear and multisensory game instructions and suitable interaction. (2) For the game context and tasks, adopt content and themes that are familiar to the user from daily life. (3) Design several different tasks corresponding to the training of various cognitive capabilities; these tasks should be easy to complete and provide practice modes. (4) Provide feedback on the training consequences. (5) Engage users and stakeholders to evaluate the design and its effects. This study proposes that for elderly adults, cognitive training games that are casual, contain familiar life contexts and have a user-friendly interface are necessary and satisfying.

In terms of game devices, a mobile tablet-based training app has several advantages that are especially beneficial for training older adults. The most important benefit is that older adults perceive tablets as personal devices that they can carry with them easily; as a result, they can flexibly integrate training into everyday life (Binder et al., 2015). For example, mobile devices are easier to carry and operate than portable devices (i.e., laptops) when the elderly have to move between day-care nursing homes and their own homes. Moreover, the screen size of tablets fits older adults' visual abilities better than smartphones, especially during tasks that are complex or involve a high cognitive load, such as reading (Werner et al., 2012; Yueh et al., 2012). Because of these advantages of tablets, older adults have rapidly adopted mobile devices and expect mobile technology to flexibly support their leisure activities, social interactions and learning.

Although studies on digital games have provided valuable insight into cognitive training and also suggested design principles for the content and interactions, they have not frequently used multiple sources (such as stakeholder attitudes and qualitative and quantitative sources) for analysis, have not described the design processes of games according to users' evaluations and have seldom tested games in a realistic context. Elderly adults adopt a technological innovation when they see that it is useful in their lives (Hanson, 2010). Understanding what components of cognitive training games affect elderly users' experiences is necessary because the usability of mobile applications is of great importance.

Meanwhile, to address the need to clarify how older users' cognitive functions can be enhanced by game training in a genuine context with stakeholders, this study adopted the research method framework of design-based research (DBR) to investigate the complex phenomenon. DBR focuses on the development of hypotheses and a framework and the contribution to model formulation, rather than model estimation or validation. As a result, it can obtain a different outcome, such as new theory (Simon, 1969; Sloane and Gorard, 2003). DBR typically triangulates multiple sources and types of data to connect intended and unintended outcomes of processes of enactment, and it increases the validity of findings through its typical partnerships and iteration (The Design-Based Research Collective, 2003). DBR enables researchers to grasp problems through iterative development and places emphasis on the authenticity of the context; it also enables a clearer understanding of the goals and implications of research, resulting in maximum design optimization (Joseph, 2004; Lin et al., 2014).

In the design of an optimized and user-friendly cognitive training game for elderly people, the specific research questions that must be answered are as follows: (1) What types of cognitive training do elderly people accept in a mobile game? and (2) What components are crucial to improving or reducing the usability of a cognitive training game for elderly people? The current study involved collaboration among experts from the medical, interaction design and engineering fields, and it designed a cognitive training app for seniors in home or nursing home settings. The iterative cycles and process that were employed to develop the app are described. The needs, preferences, technological experience and cognitive deficits of older people were evaluated and considered in the design of the cognitive training app so that it would offer comprehensive support.

## Methods

In its approach to the relatively new concept of producing cognitive training games for elderly people, the present study employed DBR (Barab and Squire, 2004). The study focus was the evolution of a cognitive training game as a design artifact through iterative processes to determine both the theoretical and practical implications of the mobile game's design. First, elderly people's experience with mobile technology and their needs and interactions with the cognitive training game were investigated. Second, their perceptions of, and the cognitive load entailed by, the mobile game were subjected to a formative assessment. This formative assessment was used to determine theoretical propositions concerning aging-related cognitive training and human-computer interactions for the design of novel cognitive training supported by mobile games. The opinions of expert stakeholders were gathered and incorporated through iterative evaluations aimed at optimizing the design.

### Research design

**Figure 2:**
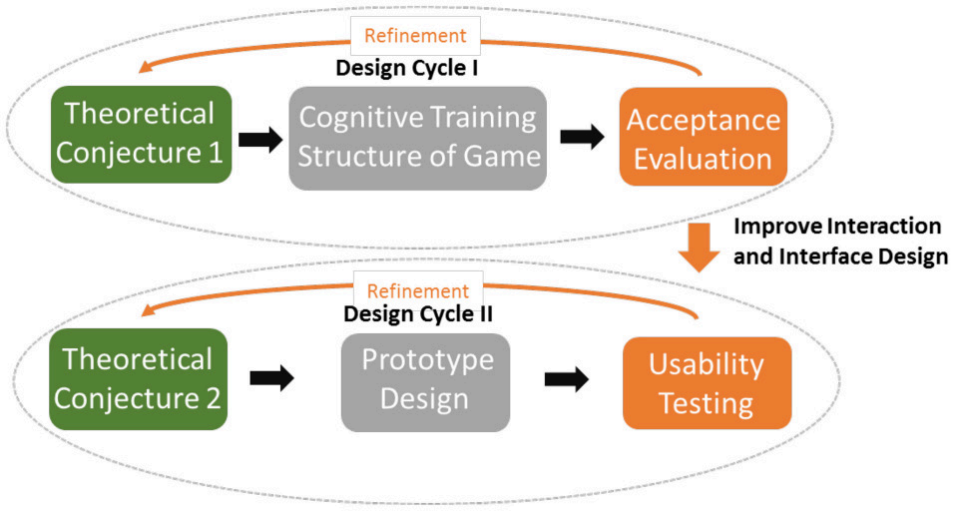
DBR process in this study.

The cognitive training game developed in this study was defined as a casual game. The game was investigated in complex real-life settings involving multiple variables, and various aspects of the design were refined using DBR. Two design cycles were adopted and are illustrated in Figure 2.

In the two iterations, successive refinements of the design, perception, analysis and redesign were made. The first iteration focused on evaluating whether the game environment with the cognitive training structure proposed in Figure 1 was accepted and also explored suggestions regarding its redesign to improve its usability.

The second iteration focused on developing and testing the form and function of the prototype to verify and refine the context and usability of the game. An acceptance evaluation and usability testing were executed at elderly participants' homes so that the data would be collected in authentic usage contexts.

### Participant recruitment

This study employed purposeful and convenience sampling methods to recruit participants. The participants included elderly people (older than 60 years) as target users who were homebound and those who received care either during the day or around the clock in a nursing home. Elderly people were recruited to perform heuristic evaluations of the mobile cognitive training app. Heuristic evaluation allows researchers to employ a small set of evaluators (three to five evaluators) to examine the interface and judge its compliance with recognized usability principles (Nielsen and Molich, 1990; Nielsen, 1994). Moreover, medical experts, interaction designers and engineering programmers were also recruited as stakeholders to provide professional suggestions on the designs of cognitive training content and interactions.

In the first iteration, four female target users, who had age-related memory decline and lived in a day-care or full-day nursing home, were recruited to participate (Table 1). They were aged from 73 to 90 years (mean = 82.75, *SD* = 7.27). All had experience with using mobile technology (smartphones and tablets), and three had their own mobile devices. They were interviewed and asked to evaluate current mobile games related to the cognitive training structure (Figure 1). These mobile games were chosen on the basis of their cognitive training structure, as determined by stakeholders and four experts, including interaction and user interface designers and programming engineers (Table 2, S-2–S-5). Game design suggestions were also collected from a medically proficient manager of a day-care nursing home (Table 2, S-1). All professional stakeholders participating in this study had more than 3 years of experience in their fields.

**Table 1:** Demographics of elderly participants.

**Design iteration**	**Participant**	**Age**	**Gender**	**Mobile technology experience**	**Own mobile device**	**Living arrangement**
Cycle I	U-1	82	F	✔	No	Full-day nursing home
	U-2	90	F	✔	Enhanced phone	Day-care nursing home
	U-3	73	F	✔	Smartphone, tablet	Day-care nursing home
	U-4	86	F	✔	Enhanced phone	Day-care nursing home
Cycle II	U-5	84	F	✔	No	Living with family
	U-6	74	M	✘	Enhanced phone	Living with family
	U-7	66	F	✘	No	Living with family
	U-8	63	F	✘	Enhanced phone	Three generation family
	U-9	61	M	✔	Smartphone	Living with spouse

*Mobile technology experience, experiences of using smartphone or tablet; F, female; M, male*.

**Table 2:** Demographics of stakeholders.

**Design iteration**	**Participant**	**Gender**	**Profession**	**Years of professional experience**
Cycle I	S-1	M	Medically proficient manager	More than 5 years
			social worker	More than 10 years
	S-2	F	Human-computer interaction and user interface design	More than 3 years
	S-3	F	Human-computer interaction design	
	S-4	M	Human-computer interaction design and programmer	
	S-5	M	Human-computer interaction design and programmer	

In the second iteration, five older adults who lived in their own homes participated and tested the usability of the prototype (Table 1). They were aged from 61 to 84 years (mean = 69.60, *SD* = 9.45). Two had experience with using mobile technology (smartphones and tablets), and three had their own mobile devices.

Every participant in this study was asked to sign an informed consent form before participating in the research. They were fully informed about the study purpose, research process, data recording, potential risk and relevant compensation. No additional ethical approval was required in this study, in accordance with national and institutional requirements.

### Procedures

The procedures of the DBR process used in this study are explained as follows.

#### First iteration

The first iteration involved the following steps:

1. Theoretical conjecture 1: Theoretical propositions for the cognitive training game structure were considered during an interpretation of the needs of elderly users of cognitive training games by researchers. The medical expert (S-1) was interviewed and his suggestions on the cognitive training structure of the game were collected.
2. Cognitive training structure of the game: Interaction designers (S-2–S-5) were recruited to help select evaluations of some current mobile games that perform cognitive training of attention, executive function, memory, language and visuospatial function and involve the completion of discrimination, visuomotor, respelling and calculation tasks.
3. Acceptance evaluation: The current mobile games with cognitive training structures selected in step 2 were presented to the participants (U-1–U-4). The elderly users were asked to complete the game tasks, and their usage behaviors and general feedback were documented.

Design cycle I was based on a theoretical review of the aging cognition and technology, and a user evaluation; the cognitive training structure of the game was optimized accordingly. The design principles, game tasks and training goals of the cognitive training game became clearer during this stage. The results of the evaluation were used to refine the initial conjecture and thus supported the second design cycle.

#### Second iteration

The second iteration involved the following steps:

1. Theoretical conjecture 2: In the second iteration, human-computer interaction theories were reviewed to compose the theoretical conjecture asserting that contexts and a usable interface are crucial components in the design of a cognitive training game for elderly users.
2. Prototype design: A prototype of the cognitive training game was developed; it incorporated interaction, task and daily life contexts according to the second theoretical conjecture and the results of the first iteration. The working prototype was then tested in authentic contexts at the participants' homes.
3. Usability evaluation: Real users (U-5–U-9) with different perspectives were recruited for a formative assessment of the mobile game in genuine home and full-day nursing home contexts. Their activities were documented and investigated. They were asked to complete the NASA Task Load Index (NASA-TLX) questionnaire to evaluate the cognitive load of the game. These evaluation results were used to identify successes and failures and to plan further improvements.

During the second iteration, the focus of the formative assessment was on the game process and cognitive load of the cognitive training game. The interaction and functions of the mobile game were modified, which completed the development of the game.

### Data collection and analysis

#### Interview of cognitive training structure of the game

To develop a cognitive training game, the theoretical bases of aging cognition and technology were reviewed and organized into design guidelines. The cognitive training structure of the game was first proposed on the basis of the presented literature review (Figure 1) and then suggested by a medical expert (S-1). A semistructured interview was conducted to acquire a professional stakeholder's (S-1) suggestions regarding the cognitive training structure of the game. The guidelines of the interview covered four aspects: (1) What are the everyday life and physical and mental statuses of elderly people in day-care nursing homes? (2) What cognitive training activities can residents participate in when in day-care nursing homes? (3) What experience do elderly people have of using technology, and what is their technological ability? (4) What is important in the design of a cognitive training game for elderly users?

This study adopted content analysis to analyze the interview document (Strauss and Corbin, 1990; Ericsson and Simon, 1993). First, the protocol analysis was employed to classify the content of interview transcripts into the categories of cognitive training activities, specific-domain cognitive training, older adults' attitudes toward and feedback on cognitive training activities, older adults' technology experiences and the expectations of the cognitive training game. Second, the classified contents were encoded. Third, the encoded contents were categorized and conceptualized. The process of encoding and categorizing were agreed upon by the three researchers.

#### Acceptance evaluation

An acceptance evaluation was conducted to examine whether or not the cognitive training structure of the game (Figure 1) was suitable for elderly people. First, the evaluation content of this structure had to be selected. The researchers and experts, i.e., designers and programmers (S-2–S-5), selected the current mobile game-dementia prevention self-diagnosis test as the evaluation content. This mobile game is relevant to various specific domains of cognition in the cognitive training structure of the game (Figure 1). However, this mobile game lacks visuomotor and respelling tasks, so the designers suggested designing simple interactions such as drawing and card reading activities to be included (Table 3). Second, the participants (U-1–U-4) were asked to complete discrimination, calculation, visuomotor and reading tasks in the games. Third, the researchers documented the users' usage behaviors while playing the game. Finally, the participants' attitudes toward and preferences of the games were collected.

**Table 3:** Contents of acceptance evaluation.

**Task**	**Description**	**Interface**	**Cognitive training domain**
Cube counting	Input the number of cubes	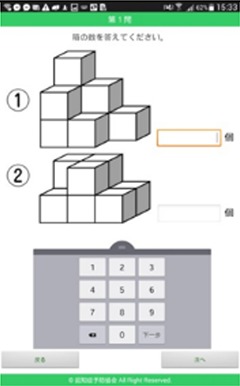	Attention executive function memory visuospatial function
Time discrimination	Input the item number corresponding to the assigned time	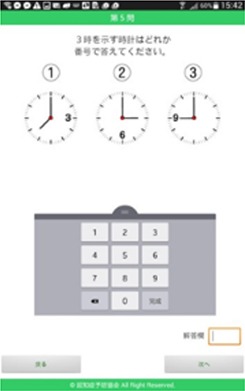	Attention executive function memory visuospatial function
Amount comparison	Input the set number that contains the most money	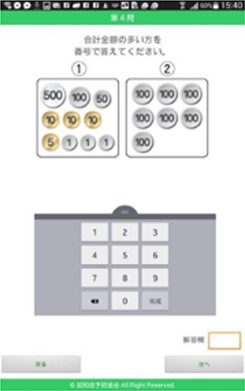	Attention executive function memory visuospatial function
Drawing	Draw the same shape	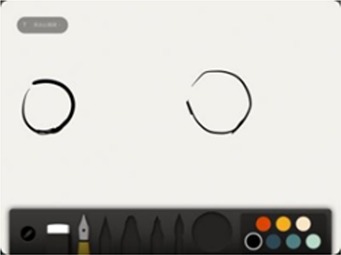	Attention executive function memory visuospatial function
Reading cards	Read aloud the cards on the tablet and respond to the cards' questions	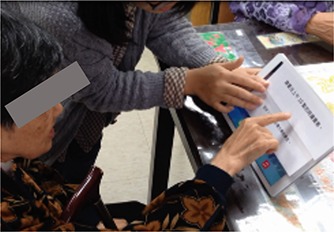	Attention executive function memory language visuospatial function

The usage behavior of game playing was recorded and analyzed according to hierarchical task analysis (HTA) of each game trial. HTA is a structural method for describing how users complete tasks through hierarchical action plans and executions (Reagan and Kidd, 2013). HTA comprises redescription and decomposition. A primary task can be transferred into a structural model with these two important elements. The former helps designers identify the primary goal and break it down into several sub-goals with various levels until they reach the expected criteria. The latter helps designers to examine every sub-goal in order, such as inputting, receiving feedback, completion time and recording errors (Shepherd, 1998).

#### Formative assessment and usability testing

A formative assessment was performed to collect feedback from the representative users and thus further the context and usability considerations of the cognitive training game. The users (U-5–U-9) played the game, completed the NASA-TLX questionnaire and were then interviewed on their general opinions of the game. The NASA-TLX questionnaire includes six load indices—mental demand, physical demand, temporal demand, performance, effort and frustration—measured using a 10-point Likert-type scale (1 = *strongly disagree*; 10 = *strongly agree*) (Hart and Staveland, 1988) and analyzed with descriptive statistics.

When the users played the game, the researchers observed and documented participants' behaviors and feedback to evaluate the interaction design. The usage behavior was recorded and analyzed according to HTA of each game trial. The time spent on each game trial was also recorded.

## Results

### Expert interview and confirmation of theoretical propositions of cognitive training game structure

To design appropriate game tasks for cognitive training, this study interviewed a professional manager (S-1) of a day-care nursing home. His suggestions were adopted to confirm the theoretical propositions of the cognitive training game structure. He also provided his observations of the elderly adults' attitudes toward the cognitive training technology.

**Figure 3:**
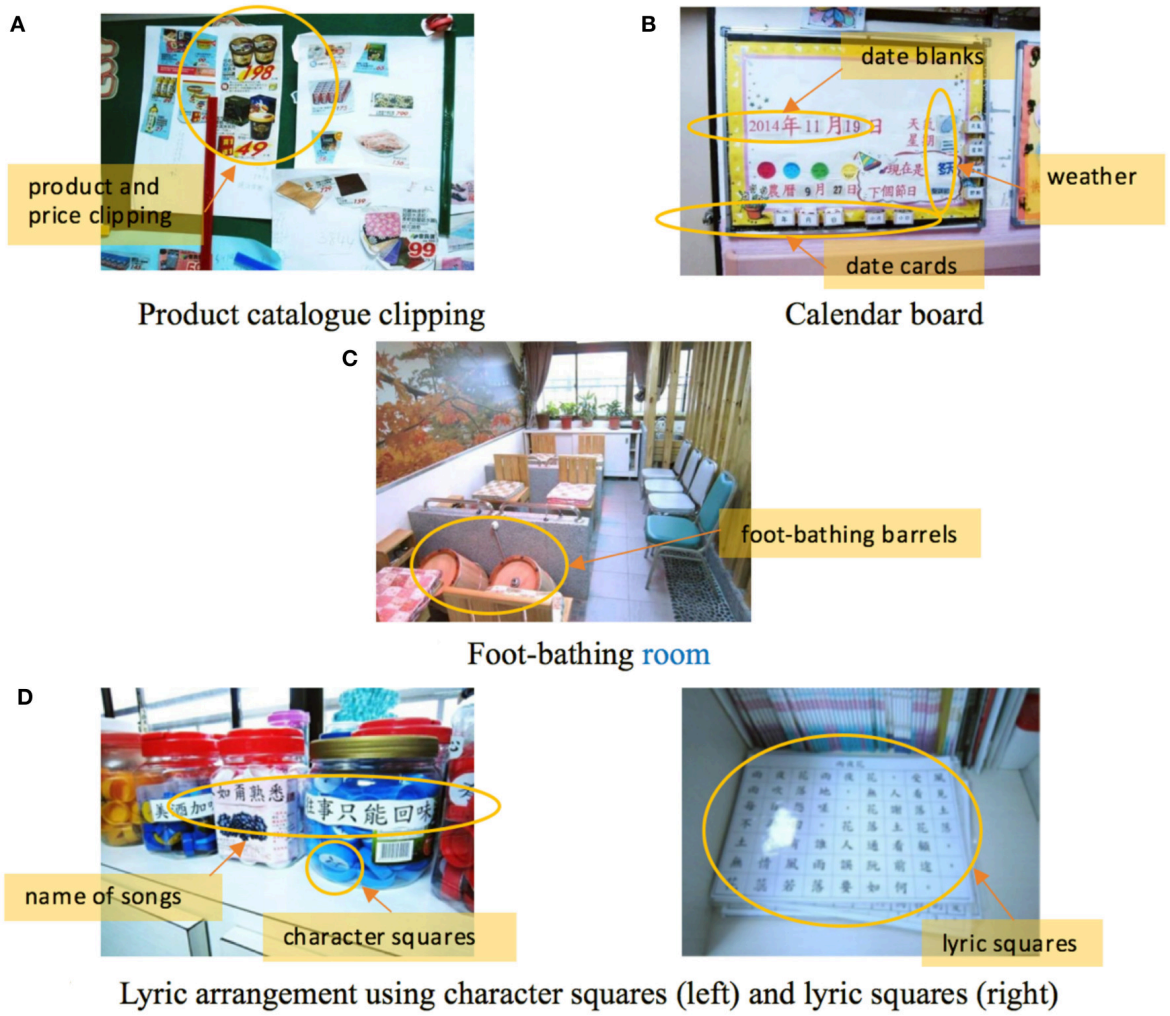
Cognitive training activities in the day-care nursing home. **(A)** Product catalog clipping **(B)** Calendar board **(C)** Foot-bathing room **(D)** Lyric arrangement using character squares (left) and Lyric squares (right).

Table 4 presents the cognitive training activities and tasks performed in the day-care nursing home that were suggested by the manager. All of the activities and tasks corresponded to associated specific-domain cognitive training of the conjectural structure (Figure 1). The easy and free creation activities such as drawing and clipping facilitated the cognition, accomplishment, activity engagement and emotional stability of elderly people. For example, Figure 3A shows the works of clipping. The elderly participants clipped the products from product catalogs to show what they wanted to buy when they imagined shopping contexts. The social interaction activities with multisensory stimulation promoted their attention, memory and language abilities, such as during karaoke and foot-bathing (Figure 3C). Certain complex activities such as the folding of balloons and handicrafts aimed to facilitate the cognitive functions of attention, executive function, memory and visuospatial function. However, older adults may perform worse on these tasks because such tasks require the execution of a complicated sequence of steps. On the other hand, the computer-based game with the simulation of an authentic context, such as baking a cake, was considered easier and was accepted by the elderly participants. As shown in Table 4, the conjectural structure was sufficient to develop a cognitive training game. The current and easy cognitive training activities were appropriate for use as training content in the game.

**Table 4:** Cognitive training activities and effectiveness in the day-care nursing home.

**Activity**	**Task**	**Stimulated cognitive constructs**				
		**Attention**	**Executive function**	**Memory**	**Language**	**Visuospatial function**
Drawing and clipping	Create paintings by free drawing and clipping	v	v			v
Karaoke	Sing after arranging lyrics using character squares (see Figure 3D)	v	v	v	v	
Lyrics arrangement using character squares		v	v			v
Outdoor trip	Interact and communicate with companions (see Figure 3C)	v	v	v	v	
Foot-bathing		v	v	v	v	
Product catalog clipping	Calculate costs in a simulated shopping context (see Figure 3A)	v	v	v		
Calendar board	Change cards indicating the date, season, weather and holiday (see Figure 3B)	v	v	v		
Making cake	A computer game guiding people to bake a cake step by step	v	v	v		v
Folding of balloons	Accomplish the task by following numerous steps	v	v	v		v
Handicrafts		v	v	v		v

In terms of the expert's attitude toward the cognitive training game, he specifically suggested four principles for the design of the cognitive training game for elderly people: (1) The game should train social interaction and life function capabilities; (2) the content or tasks should be associated with users' life experience; (3) the game should provide multisensory stimulation, including lights, sounds and colors; and (4) the game tasks should give users a sense of accomplishment to motivate them to play the game regularly.

### Users' acceptance evaluation of cognitive training games

According to the theories of aging cognition, this study proposes the cognitive training structure of a game, which includes discrimination, visuomotor, respelling and calculation tasks. These tasks are intended to train various cognitive aspects in elderly users: attention, executive function, memory, language and visuospatial function (Figure 1). The identification of this structure was followed by the selection of current cognitive training games (Table 3) to investigate users' acceptance in cycle I.

As demonstrated in Table 5, tasks related to mental rotation and counting were perceived as more difficult by the participants. Their failure in these two games mainly resulted from the inappropriate content and interface designs. The participants responded that they preferred larger icons and words (26 pt). They also suggested that the input function should be more intuitive. For example, in the game of time discrimination, they expected to respond by tapping the clock directly, rather than by using the number keyboard.

**Table 5:** Failure and difficulty evaluation of the current cognitive training games.

**Task**	**Cognitive training domain**	**Failure evaluation**	**Difficulty evaluation**
Cube counting	Attention executive function memory visuospatial function	•The hidden cubes were difficult to discriminate •The icons were too small to read and tap	Difficult
Time discrimination	Attention executive function memory Visuospatial function	•Input the answer by tapping the clock	Easy
Amount comparison	Attention executive function memory visuospatial function	•Incorrect counting •The coin icons were too small to discriminate	Difficult
Drawing	Attention executive function memory visuospatial function	•No failure	Easy
Reading cards	Attention executive function memory language visuospatial function	•No failure	Easy

Although some game tasks were difficult and the interface and interaction designs were not sufficiently appropriate, every participant perceived the game as interesting. They were willing to use these types of mobile games in the future. Therefore, the cognitive training structure of the game proposed by this study was accepted by the elderly participants. However, some modification of the tasks and interaction design were required to improve the experience and performance.

### Game design: refinement and development of the cognitive training game

The consensus among the elderly users and the stakeholder's evaluation were used to support, validate and refine the design structure of the cognitive training game. The game comprised not only the cognitive training structure but also experiential contexts, clear and concise instructions, and multisensory and intuitive interaction with immediate feedback to improve the acceptance, experience and performance. Following this optimized game structure, a working prototype was developed. The prototype consisted of game tasks involving suitable experiential contexts and a user-friendly interface and interaction.

#### Game tasks, contexts, and cognitive training

The working prototype, named Brain Win, had a functional appearance and was designed to involve four types of cognitive tasks—discrimination, visuomotor, respelling and calculation tasks—in six game contexts that were connected with the life experience of older adults. The game contexts and elements were designed according to the interview with the manager of the day-care nursing home (Table 4 and Figure 3) and the preferred leisure activities of elderly people, including shopping, singing, playing chess and mahjong, taking a walk, and accompanying and playing with family and grandchildren in particular (Shi et al., 2000; Chou et al., 2004; Lee et al., 2007). The tasks corresponded to different types of domain-specific cognitive training (Table 6).

**Table 6:** Game tasks, contexts and stimulated cognitive constructs.

**Type of task**	**Context theme**	**Stimulated cognitive constructs**				
		**Attention**	**Executive function**	**Memory**	**Language**	**Visuospatial function**
Discrimination	My calendar	v	v	v		
	Finding object during a phone call	v	v	v	v	
	Go to the zoo	v	v	v		
Visuomotor	Go to the market	v	v	v		v
Respelling	Super singer	v	v	v	v	v
Calculation	Shopping in the market	v	v	v		

**Figure 4:**
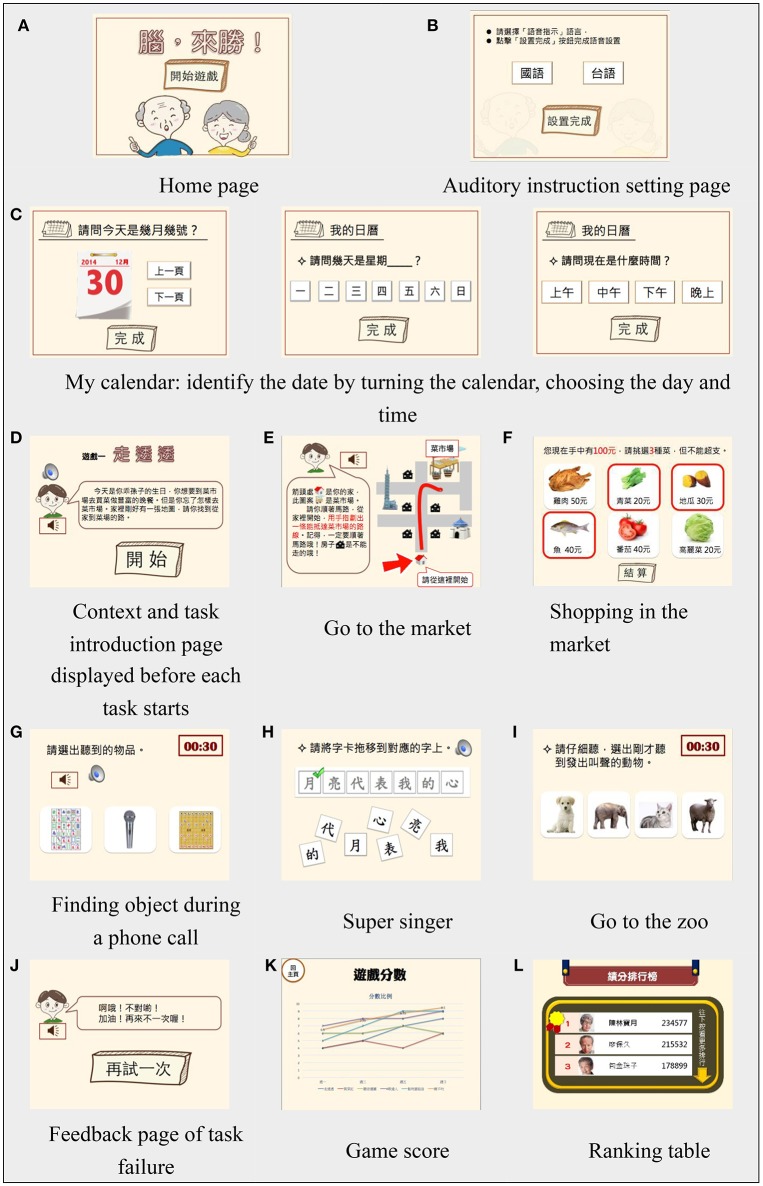
User interface prototype. **(A)** Home page **(B)** Auditory instruction setting page **(C)** My calendar: identify the date by turning the calendar, choosing the day and time **(D)** Context and task introduction page displayed before each task starts **(E)** Go to the market **(F)** Shopping in the market **(G)** Finding object during a phone call **(H)** Super singer **(I)** Go to the zoo **(J)** Feedback page of task failure **(K)** Game score **(L)** Ranking table.

The following is a specific explanation of the game tasks and contexts, in the order that they are encountered in the game:

1. My calendar: Participants had to identify the date by turning the calendar and selecting the correct day and time. These tasks made the participants orientate themselves in real time using discrimination tasks that involved the cognitive operations of attention, executive function and memory (Figure 4C).
2. Go to market: The game specified that the participants wanted to prepare dinner for their grandson's birthday and had to go to the market to purchase ingredients (Figure 4D). The participants had to read the map and draw the route to the market. This task trained the participants' attention, executive function, memory and especially visuospatial function (Figure 4E).
3. Shopping in the market: Following on from the previous context, the participants had to buy three ingredients with a budget of NT$100. During this task, the participants had to concentrate on the information icons and remember the costs of their selected items to perform cumulative calculations. This task thus trained participants' cognitive functions of attention, memory and executive function (Figure 4F).
4. Finding object during a phone call: In this game context, the participants received a phone call from their son asking them to find something in his bedroom. Participants had to listen for the target word and select the corresponding icon. The auditory cue trained participants to search their semantic memory and retrieve the corresponding icon. This task trained the participants' cognitive functions of attention, memory, language, and executive function (Figure 4G).
5. Super singer: In this context, the participants went to karaoke with a friend and had to reorganize the character cards containing song lyrics. The true lyrics were presented, and participants had to move the character cards onto them. The task was performed with musical accompaniment of the song described by the lyrics, which enabled semantic memory lyric retrieval. Participants could also recognize characters by comparing their shapes. This task trained the participants' cognitive functions of attention, memory, language, visuospatial function, and executive function (Figure 4H).
6. Go to the zoo: In this game, the participants went to the zoo with their grandsons and had to introduce animals to the grandsons after two animals made characteristic noises. Participants had to remember the noises and select the corresponding animals. This task trained participants' cognitive functions of attention, memory and executive function (Figure 4I).

Once each task was completed, a feedback page indicating success and failure was displayed with applause sounds and encouragement, respectively (Figure 4J). When participants successfully completed tasks, they could choose to practice the particular game, check their game scores (Figure 4K), or check their positions in the ranking table (Figure 4L).

#### Interface and interaction

The game instruction was supported by visual and auditory design elements. The task explanations were concise and displayed in a font size of at least 26 pt. To decrease the working memory load, each task instruction was displayed continuously during the task until the user made choices. The auditory instructions were read in the Mandarin and Taiwanese dialects to help users retrieve their semantic memory to ensure that they could comprehend the instructions in a language that was familiar to them (Figure 4B).

The buttons and icons used had a realistic or skeuomorphic appearance so that elderly users could connect them to their life experience. For instance, when users were required to buy food items, the vegetable and meat icons were all realistic pictures (Figure 4F). Additionally, the buttons were all raised shapes. The effective touch range of the buttons and icons extended more than 20 mm on each side to accommodate the decreased visual perception and finger sensitivity of older adults. The button and icon interaction cues were color changes and sounds. The first button in the game was designed to flash to prompt users to tap, recognize and memorize the input button (Figure 4A). Then, when users tapped the button, it flashed and made a feedback sound.

Unlike existing research on the interaction design of digital games, the present study investigated the design more specifically through exhaustive and iterative user evaluations. Providing a user-friendly interface can increase the willingness of elderly people to adopt new technology. A satisfying interaction experience also increases the likelihood that they will continue using the game. A formative assessment was finally conducted to confirm the usability of the game.

### Results of the confirmation test

The working prototype with complete functionality was presented to five users for a formative assessment in the actual settings of the users' homes. The participants (U-5–U-9) were three females and two males from 61 to 84 years old. The confirmation test focused on the usability of the game. All five participants completed the six game tasks before answering the NASA-TLX questionnaire and expressing their general attitudes toward the game.

**Figure 5:**
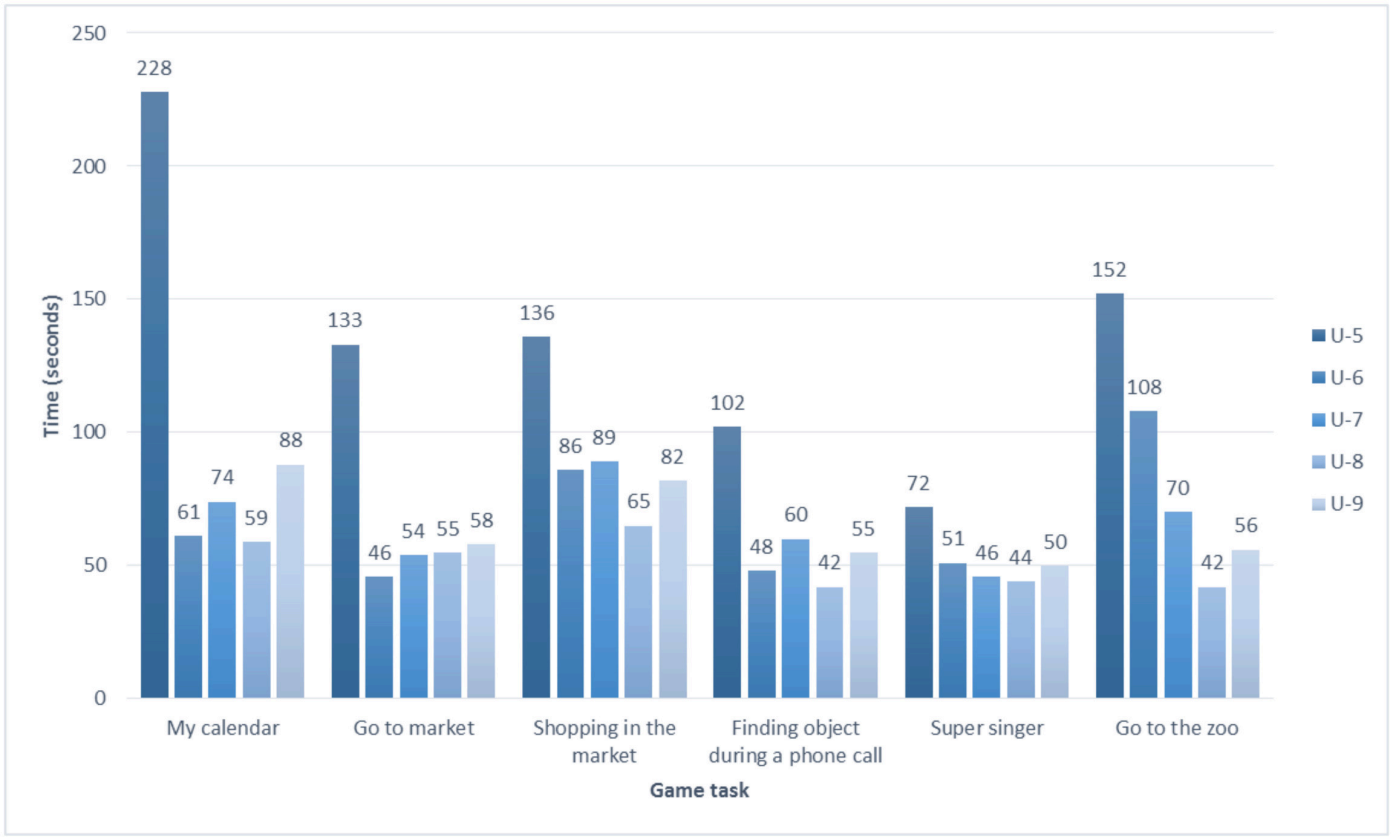
Game task completion times per game and per user.

Their performances on the six game tasks are shown in Table 7 and Figure 5. The average time taken to complete the six game tasks was 462.40 seconds (*SD* = 205.11). Three game tasks were more difficult, namely, My calendar (mean = 102.00, *SD* = 71.39), Shopping in the market (mean = 91.60, *SD* = 26.50) and Go to the zoo (mean = 85.60, *SD* = 44.53). The participant older than 80 years (U-5) had to spend over twice the time taken by the younger participants to complete all the game tasks.

**Table 7:** Completion time of game task (seconds).

**Game task**	**U-5**	**U-6**	**U-7**	**U-8**	**U-9**	**Mean**	***SD***
My calendar	228	61	74	59	88	102.00	71.39
Go to market	133	46	54	55	58	69.20	35.94
Shopping in the market	136	86	89	65	82	91.60	26.50
Finding object during a phone call	102	48	60	42	55	61.40	23.70
Super singer	72	51	46	44	50	52.60	11.22
Go to the zoo	152	108	70	42	56	85.60	44.53
Total time	823	400	393	307	389	462.40	205.11

According to HTA, the failure manipulations and longer completion times of My calendar, Shopping in the market and Go to the zoo could be investigated and elaborated. In terms of the game contents, My calendar entailed the highest number of manipulation steps. The participants could not stop tapping the icon for turning the calendar until the date was right, and then they had to input the right day and time period. Therefore, participants had to spend more time to complete these steps. Shopping in the market required participants to calculate the accumulated amount of money of the chosen products, so this task demanded more integrative cognitive resources. Every participant presented thinking expressions and thought aloud about the calculations when tapping the product icons. Go to the zoo asked the participants to remember two successive animal sounds, so the participants tended to forget (U-6) or needed more time to recall the second animal sound.

On the other hand, the interface and interaction designs affected the performance of the game tasks. During the assessment, the participant aged 84 years (U-5) responded that she usually forgot the task instructions and ignored the task count button in Shopping in the market (Figure 4F). Two participants (U-5 and U-7) were confused by the task of turning the calendar in “My calendar” (Figure 4C) because they tried to turn the calendar by tapping the calendar directly rather than by tapping the icons for moving to the previous or next page.

As shown in Table 8, the participants generally felt satisfied with their performances (mean = 8.80, *SD* = 0.84); more importantly, they did not find the games frustrating (mean = 1.20, *SD* = 0.54). The participants thought the games to be slightly physically demanding (mean = 3.64, *SD* = 1.82) and mentally demanding (mean = 4.00, *SD* = 3.74). To train the cognitive function of elderly people, games should require low levels of physical and mental demand. Generally, the participants were willing to use the cognitive training game and had positive attitudes toward the game. The participant aged 84 years suggested that the instructions of manipulation should be more salient. On the other hand, the participants who were younger than 80 years considered the game to be easy and expected the more difficult levels and virtual money or food as rewards for training.

**Table 8:** Cognitive load evaluation based on NASA-TLX.

**Items**	**Mean**	***SD***
Mental demand	4.00	3.74
Physical demand	3.60	1.82
Temporal demand	1.80	0.84
Performance	8.80	0.84
Effort	2.20	1.30
Frustration	1.20	0.45

**Figure 6:**
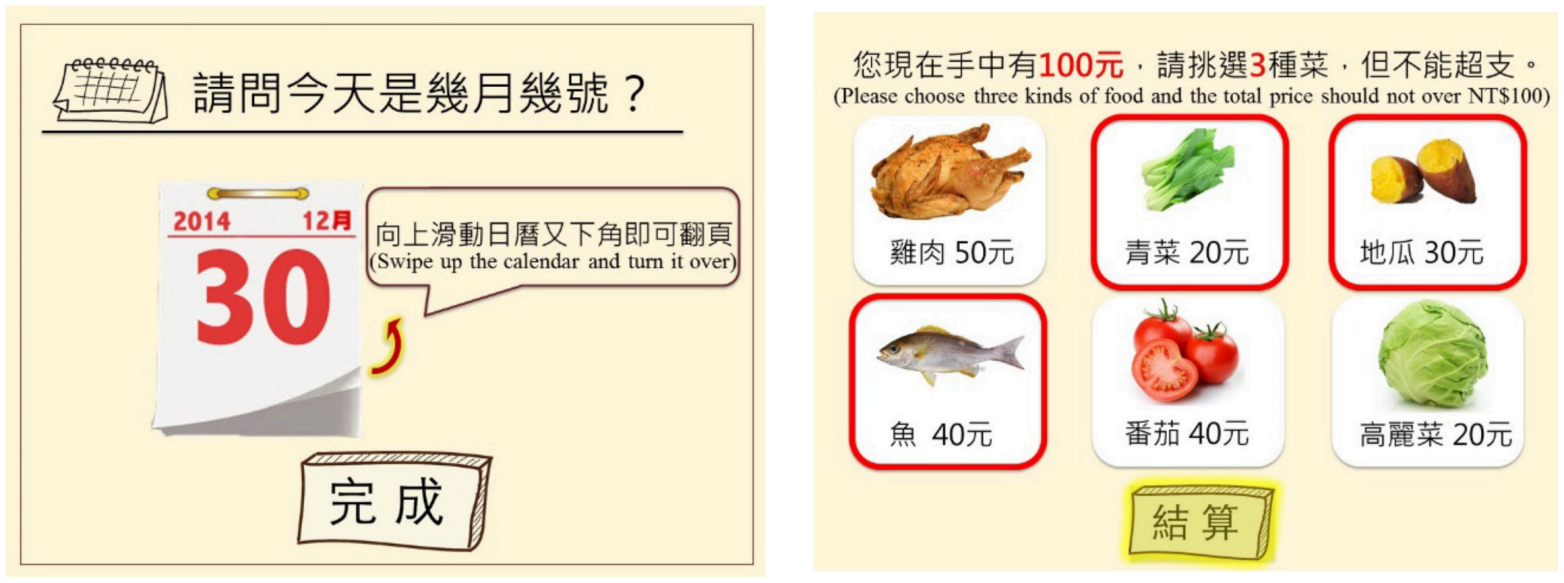
Refined interfaces of My calendar and Shopping in the market.

According to the results, the contexts and tasks of the game were generally satisfying and acceptable. Interface and interaction problems were encountered by users for the My calendar and Shopping in the market tasks, so these were modified. As shown in Figure 6, the mode of calendar turning was changed from button tapping to swiping, which is more intuitive, and this mode was indicated by the textual instruction and a flashing arrow. The count button in Shopping in the market was modified to flash with a yellow light to attract the user's attention after he or she had chosen three ingredients.

## Discussion and conclusion

This study aimed to develop an improved design for game-based cognitive training for seniors using mobile devices. The Design Based Research method was employed to answer the research questions, including: (1) What types of cognitive training do elderly people accept in a mobile game? and (2) What components are crucial to improving or reducing the usability of a cognitive training game for elderly people?

According to the results of expert interviews and the acceptance evaluation in the first iterative cycle, the theoretical propositions concerning aging-related cognitive training (Figure 1) in the design of novel cognitive training supported by mobile games was confirmed. Both medical expert and elderly participants accepted the cognitive training structure of the game. The effectiveness of cognitive training activities for elderly people in day-care nursing homes and suggestions from the manager agreed with the reviews of aging cognition theory and interaction design for the elderly (Ijsselsteijn et al., 2007; Schmiedek et al., 2010; Trefry, 2010; Lu and Yueh, 2015). Specifically, the design of the experiential context, social interaction and positive feedback should be emphasized. Therefore, lyrics arrangement using character squares (Figure 3D), product catalog clipping (Figure 3A) and calendar board changing (Figure 3B) were suitable contexts for the mobile game design of this study, for they corresponded to the leisure activities of older adults, such as karaoke and shopping (Shi et al., 2000; Chou et al., 2004). They also echoed the domain-specific cognitive training and tasks in the conjectured game structure and were neither too easy nor too difficult.

The results of the evaluation echoed the theories of aging technology design. First, the content of a mobile game should be large, such as its font size, icons, buttons and tap area (Charness and Jastrzembki, 2009; Fisk et al., 2009; Yueh et al., 2013, 2014). For instance, the effective touch range of buttons should extend at least 20 mm per side in mobile games designed for elderly adults (Siek et al., 2005), and the font size should be at least 26 pt. Second, the operation should be intuitive and accompanied by multisensory cues and feedback (Lee and Kuo, 2001; Harada et al., 2013). Third, accomplishing the game's tasks should require a low amount of working memory, and auditory cues should be provided to facilitate the users' retrieval of semantic memory (Ferreira and Pithan, 2005). Fourth, the cognitive abilities of attention, executive function, memory, language and visuospatial function decline with age, so the entire design should be clear, simple, and intuitive, and suitable feedback should be provided (Fisk et al., 2009; Drag and Bieliauskas, 2010; Lu and Yueh, 2015). The results of the evaluation enabled interpretation and refinement of the theoretical propositions and identified related needs and problems before the development of a form-study prototype and further interviews in the next iteration.

In the second iterative cycle, the results in the first iterative cycle supported and supplemented the theoretical propositions concerning training contexts and the importance of a usable interface in the design of a cognitive training game for elderly users. Therefore, a working prototype that conformed to the needs of older adults in terms of form, appearance and working function was developed. Afterwards, the usability evaluation was conducted to confirm the design of both the training contexts and interface to explore what components are crucial to the game. The results showed that elderly users generally perceived that low effort was needed to complete the game and approved of their own performance. However, game tasks with many manipulation steps and complex integrative cognitive functions required more time to work through. That is, older adults' performances of attention, executive function and memory are worse when they perform complex tasks (Dobbs and Rule, 1989; Lee and Kuo, 2001; Wang et al., 2011). Furthermore, the instruction and feedback design should be more salient to guide users on how to interact with the game. A more intuitive interaction design with authentic contexts can better improve performance (Fisk et al., 2009; Drag and Bieliauskas, 2010; Lu and Yueh, 2015). Finally, the optimized interface of the My calendar and Shopping in the market tasks were proposed in line with the results of the usability evaluation.

Based on the results, this study offers design suggestions, theoretical contributions, limitations and further issues deserving future research. In terms of design suggestions, this study differed from previous studies of game-based cognitive training for elderly people (Van Muijden et al., 2012; Binder et al., 2015) in that it not only included training tasks based on theories of aging cognition but also concentrated on the game's interface and interaction design to ensure that the game would be easy, user-friendly, satisfying and relatable to the user's everyday life. The design suggestions are offered to increase the adoption and self-perceived performance of elderly people using casual cognitive training games. First, the cognitive training structure of the game must be relatable to the users' life experience if the training tasks are to be accepted. Second, the interface of and interaction within the game should correspond to familiar elements and operations in the lives of elderly people so that the interaction will be intuitive. Third, the instructions and feedback in the game must be multisensory because such input improves user performance.

This study employed a design-based approach with an aim to improve the standards of research on the use of mobile games as a suitable intervention for cognitive training of elderly people. In addition, the iterative design process was also documented in authentic contexts. Through this procedure, this study derived the following conclusions, which are crucial to the application of DBR in conducting research. First, research should involve interdisciplinary collaboration, including at least academics from the fields of interaction design and engineer science, mechanism experts and stakeholders, if the insufficiencies of conventional designer-driven methods are to be overcome. Second, theory-driven intervention and conjectures must be verified and refined for authentic contexts to solve specific research problems and improve designs.

This study also has some limitations. The cognitive training game developed in this study was user-friendly, but such games can be addictive and may have a negative effect on user health. In addition, if mobile games are implemented in nursing homes, management strategies must be devised to avoid problems regarding ownership and usage time. Finally, this study was limited by resource availability, including accessibility to institutions and users, the system stability of the prototype and the efficiency of cognitive training. It is suggested that the effectiveness of cognitive training in mobile games be evaluated in the future through field and physical testing on a larger scale involving more elderly users, and that the research be conducted in natural settings. The next task for this research is to incorporate theoretical propositions regarding the interactions among elderly people, mobile games and cognitive training evaluation.

## Author contributions

ML, WL, and HY developed the concept and design of this study. Data was collected and analyzed by ML. All authors interpreted the results. ML drafted the manuscript under the supervision of WL and HY, and WL and HY provided critical revisions. All authors approved the final version of the manuscript for submission.

### Conflict of interest statement

The authors declare that the research was conducted in the absence of any commercial or financial relationships that could be construed as a potential conflict of interest.
